# 43.4 THE ROLE OF MOLECULAR IMAGING IN GUIDING DRUG DEVELOPMENT

**DOI:** 10.1093/schbul/sby014.182

**Published:** 2018-04-01

**Authors:** Anissa Abi-Dargham, Lawrence Kegeles, Mark Slifstein

**Affiliations:** 1 Stony Brook University School of Medicine; 2 Columbia University & New York State Psychiatric Institute; 3 Stony Brook University

## Abstract

**Background:**

Molecular imaging with Positron Emission Tomography (PET) has the unique capability of examining molecules in the brains of human subjects in real time. This has been well exploited in the study of dopaminergic transmission in schizophrenia. The results have on one hand confirmed some predictions, and on the other hand they have brought up unexpected findings, that have the potential to affect drug development in a major way.

**Methods:**

PET studies of dopamine release, synthesis, storage, receptors and transporters across labs and different experiments will be reviewed to extract findings that differ from preconceived notions. These have the capability of re-focusing drug development based on new evidence.

**Results:**

Expected findings that PET studies have confirmed are those of striatal dopaminergic excess, measured as enhanced presynaptic release capacity and presynaptic enzymatic synthesis capacity. Also expected was hypodopaminergic transmission in the dorso-lateral prefrontal cortex.

The unexpected findings from PET have been as follows: 1) the striatal excess is most predominant in the associative striatum, rather than the limbic striatum. 2) hypodopaminergia is generalized to all extrastriatal areas of the brain

These observations of opposite presynaptic dopaminergic dysfunction between striatal and extrastriatal regions suggest that the striatal excess may not be driven by activity levels of midbrain DA cells but may result from abnormal striatal local regulation of presynaptic DA release, affecting specifically the associative striatum, region which shows largest magnitude of excess DA. Furthermore alterations in dopaminergic signaling in opposing directions may have a dual effect on learning across functional domains by weakening learning from salient or relevant signals and reinforcing stimulus independent signals, thus explaining different symptom domains.

**Discussion:**

More work is needed to understand the cellular mechanisms of dopaminergic dysfunction in schizophrenia. The evidence presented here, while limited, highlights the challenges of drug development in the absence of real in vivo information from patients, and the need for multidisciplinary cross talk to address these challenges.

